# Estrogenic Modulation of Retinal Sensitivity in Reproductive Female Túngara Frogs

**DOI:** 10.1093/icb/icab032

**Published:** 2021-04-26

**Authors:** Caitlin E Leslie, Whitney Walkowski, Robert F Rosencrans, William C Gordon, Nicolas G Bazan, Michael J Ryan, Hamilton E Farris

**Affiliations:** 1 Department of Integrative Biology, University of Texas, Austin, TX 78712, USA; 2 Neuroscience Center, Louisiana State University School of Medicine, 2020 Gravier Street, New Orleans, LA 70112, USA; 3 Department of Cell Biology and Anatomy, Louisiana State University School of Medicine, New Orleans, LA 70112, USA; 4 Department of Ophthalmology, Louisiana State University School of Medicine, New Orleans, LA 70112, USA; 5 Department of Otorhinolaryngology, Louisiana State University School of Medicine, New Orleans, LA 70112, USA

## Abstract

Although mate searching behavior in female túngara frogs (*Physalaemus pustulosus*) is nocturnal and largely mediated by acoustic cues, male signaling includes visual cues produced by the vocal sac. To compensate for these low light conditions, visual sensitivity in females is modulated when they are in a reproductive state, as retinal thresholds are decreased. This study tested whether estradiol (E2) plays a role in this modulation. Female túngara frogs were injected with either human chorionic gonadotropin (hCG) or a combination of hCG and fadrozole. hCG induces a reproductive state and increases retinal sensitivity, while fadrozole is an aromatase inhibitor that blocks hCG-induced E2 synthesis. In an analysis of scotopic electroretinograms (ERGs), hCG treatment lowered the threshold for eliciting a b-wave response, whereas the addition of fadrozole abolished this effect, matching thresholds in non-reproductive saline-injected controls. This suggests that blocking E2 synthesis blocked the hCG-mediated reproductive modulation of retinal sensitivity. By implicating E2 in control of retinal sensitivity, our data add to growing evidence that the targets of gonadal steroid feedback loops include sensory receptor organs, where stimulus sensitivity may be modulated, rather than more central brain nuclei, where modulation may affect mechanisms involved in motivation.

## Introduction

The behavioral significance of stimuli is often context dependent, especially for communication signals. For example, responses to sexual signals may vary under different social, ecological, and physiological conditions (Gall and Wilczynski 2015; Lea and Ryan 2015; Reding and Cummings 2017, 2018), and in some contexts elicit no response at all (Rand et al. 1997). How might these different behavioral decisions result from the same stimuli? In signal detection theory, a change in response to stimuli is thought to be mediated either through shifts in the decision criterion and/or through changes in the sensory responses themselves (Green and Swets 1966; Alves-Pinto et al. 2012). That is, observed context-dependent responses to stimuli may be based on changes in motivation (i.e., stimulus value or “just meaningful differences”), or changes in stimulus sensitivity (“just noticeable differences”) (Lynch 2017). From a mechanistic point of view, it may be difficult to untangle these, especially in behavioral assays; experiments would need to be specifically designed to manipulate a decision criterion (Stuttgen et al. 2011; Mill et al. 2014). Furthermore, the two targets of modulation may be concurrent and interact. One approach to distinguishing how context-dependent modulation is mediated would be to assess modulation of sensory receptor organs (Sisneros et al. 2004; Coffin et al. 2012), where stimulus sensitivity is arguably more likely than motivation to be modulated. Compared with endocrine mechanisms that modulate function in the brain and spinal cord (including modulation of stimulus value or reward; Dreher et al. 2007; Micevych and Meisel 2017), there appear to be fewer data implicating sensory receptor organs as targets in modulatory endocrine feedback loops (Supplementary Table S1). While studies have shown modulation of stimulus processing in central circuitry, including in our focal taxon, frogs (Lynch and Wilczynski 2008; Chakraborty and Burmeister 2015), there is growing evidence that receptor organs are targets of modulation as well, directly changing sensitivity to stimuli (Butler et al. 2019; Leslie et al. 2020). In this study we build on previous work to investigate whether estrogenic mechanisms associated with reproductive state modulate retinal sensitivity in a subject that uses visual sexual cues.

Reproductive female túngara frogs (*Physalaemus pustulosus*) exhibit increased behavioral sensitivity to light (Cummings et al. 2008), which appears to be mediated by mechanisms in the retina (Leslie et al. 2020). However, the endocrine modulators of this phenomenon are still unknown. In previous work, modulation of retinal sensitivity was achieved with an injection of human chorionic gonadotropin (hCG) (Leslie et al. 2020), which binds to luteinizing hormone receptors (Menon and Menon 2012), thereby stimulating the gonads of both sexes to release steroid hormones into the bloodstream. One of the effects of hCG injection in female túngara frogs is an increase in the steroid hormone 17β estrogen estradiol (E2), the major female sex steroid (Lynch and Wilczynski 2006). There are numerous potential neural targets for E2 modulation of reproductive behavior, including in sensory receptor organs (Supplementary Table S1). In this study, experiments were designed to determine if E2 plays a role in mediating hCG-induced retinal sensitivity change in the túngara frog.

Estradiol is necessary for and intricately linked with female reproductive behavior in túngara frogs (for review, see Wilczynski and Lynch 2011). Plasma E2 and progesterone are elevated in females during amplexus (Lynch et al. 2005; Lynch and Wilczynski 2005). Exposure to male choruses for 10 consecutive nights significantly elevates plasma E2 concentrations in females (Lynch and Wilczynski 2006). Additionally, injections of E2 increase phonotaxis behavior and cause females to show similar call preferences to those under natural breeding conditions. While hCG injection also increases phonotaxis behavior, combining hCG with fadrozole, an aromatase inhibitor, blocks this effect (Chakraborty and Burmeister 2009), presumably by blocking aromatase conversion of testosterone to estradiol.

Evidence suggesting direct effects of estrogen on the retina has been found in several species. For example, aromatase has been found in the goldfish (*Carassius auratus*) retina, including in the inner nuclear layer (Gelinas and Callard 1993, 1997). Additionally, the gene expression of several opsins in mosquitofish and sailfin molly females increases with increased estradiol exposure (Friesen et al. 2017b). Estrogen receptors in retinas have been found in a variety of species, including humans, bovines, rats, and fish (Begay et al. 1994; Kobayashi et al. 1998; Ogueta et al. 1999; Tchoudakova et al. 1999; Mangiamele et al. 2017). To investigate the potential role of E2 in modulating retinal sensitivity, we conducted scotopic (nocturnal vision) electroretinograms (ERGs) with females injected with hCG and fadrozole. We hypothesize that if E2 is sufficient for the increased visual sensitivity seen in females injected with hCG, then inhibiting aromatase with fadrozole should block the effects of hCG, leading to unmodulated retinal thresholds which match those of control non-reproductive females.

## Materials and methods

All animal care, experiments, and analytic methods are based on our previous work on frog retinal sensitivity (Rosencrans et al. 2018; Leslie et al. 2020).

### Research animals

All experiments were approved by the Institutional Animal Care and Use Committees of the University of Texas at Austin; Louisiana State University Health Sciences Center, New Orleans; and the Smithsonian Tropical Research Institute. Subjects included laboratory-reared frogs as well as wild-caught frogs from Panama. To prevent breeding, all frogs were housed individually. The frogs were fed *ad libitum* and housed in an “a-seasonal” environment: 12:12 light/dark cycle (300 cd/m^2^), temperature (23.3°C), and humidity (>70%). Thus, there were no seasonal cues (reproductive versus dry seasons).

### Hormone treatments

In order to investigate the effects of E2 on retinal sensitivity, scotopic ERGs were conducted with female túngara frogs in three treatment groups: 1, saline-injected control (*n* = 7); 2, injected with hCG (*n* = 7); and 3, injected with a combination of hCG and fadrozole (hCG + fadrozole) (*n* = 7). The injections for the hCG + fadrozole group followed the protocol of Chakraborty and Burmeister (2009), which used fadrozole to block estradiol production in hCG injected túngara frogs. This blocking effect likely results from inhibiting aromatase (Ankley et al. 2002), an enzyme that converts androgens to estrogens in vertebrate brains and gonads (Callard et al. 1978a, 1978b). Injection protocols were as follows: Day 1: group 3 received a subcutaneous injection of a single dose of fadrozole (50 μg; Sigma–Aldrich, St. Louis, MO); groups 1 and 2 received saline. Day 2: group 3 received a second dose of fadrozole along with a dose of hCG (500 IU; Sigma) in two sequential subcutaneous injections. Group 2 received an injection of hCG (500 IU; Sigma). Group 1 received saline. All animals were then placed in dark adaptation containers (minimum 16 h). Day 3: ERGs were run 16–18 h after the last injection. Each injection was dissolved in (50 µL) saline solution (in mM): 126 NaCl, 0.5 KCl, 2.8 CaCl_2_, 2.2 MgCl_2_, and 10 NaHEPES, pH 7.4 (274 mOsm).

### Electroretinograms

All animals were dark adapted in a light-tight box for at least 16 h prior to ERG recordings. All ERG preparations after dark adaption were done under dim red light (∼650 nm). Frogs were first paralyzed with an intramuscular injection of succinylcholine chloride (15 μg/g; Sigma–Aldrich), then each eye was treated with atropine sulfate (1%) to maintain pupil dilation. Frogs were then placed on a damp towel in a dark sound booth (Industrial Acoustic Company, Inc.) lined with a Faraday cage. All light levels, including flash stimuli and background (0 cd/m^2^) were calibrated with a LI-COR light meter (Model LI-189 with photometric probe; Lincoln, NE). Stimuli were produced using a Xenon light source and power supply (Oriel Instruments), gated by a Uniblitz shutter (Model VMM-D1), and directed via light guide to illuminate the entire cornea of one eye. Subdermal needle electrodes (GRASS Technologies or Harvard Apparatus) were inserted at the base of the skull and in the leg for indifferent and ground recordings, respectively. Silver/silver-chloride electrodes placed around the corneal periphery of the stimulated eye recorded retinal responses (one eye recorded per frog). The responses were amplified (GRASS P511), filtered (1–100 Hz), and digitized (Cambridge Electronic Design 1401) for offline analysis.

Scotopic ERGs primarily test rod-dominated (nocturnal) vision: the visual condition in which hCG was found to modulate female retinal sensitivity (Leslie et al. 2020). For the scotopic procedure, following preparation under red light and prior to recording, there were 6 min of dark adaptation. Subsequently, a series of 3 ms duration light flashes were delivered at increasing light intensities (4.8 * 10^−7^ to 23.5 cd s/m^2^) with four sequential flashes at each intensity (18 steps). Intensity was controlled using neutral density filtering (Melles Griot) of the xenon source. To prevent retinal adaptation, there was no illumination between flashes. Inter-flash intervals (30–60 s) and intervals between intensity steps (60–120 s) increased as light intensity increased.

### ERG data analysis

**Fig. 1:**
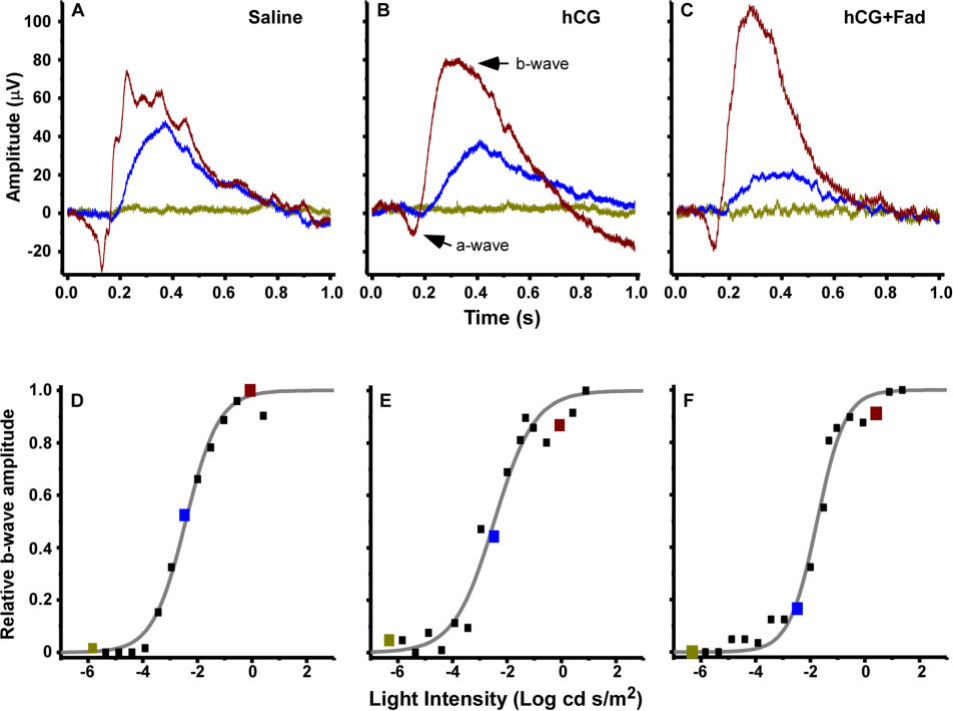
(**A–C**) Examples of raw traces from ERG recordings from single individuals in the three treatments groups. The three traces in each panel are responses to light flashes at high (red), medium (blue), and low (green) intensities. ERGs exhibited typical a- and b-wave responses (arrows for red trace in B). (**D–F**) Square symbols are the relative b-wave amplitudes as function of light intensity for the above recordings, generating V-Log(*I*) curves. Gray curves are the Boltzmann fits for each individual. Colored symbols are the relative amplitudes for the matching example traces above, illustrating b-waves near saturation (red), on the steep part of the curve (blue), and below threshold (green).

**Fig. 2:**
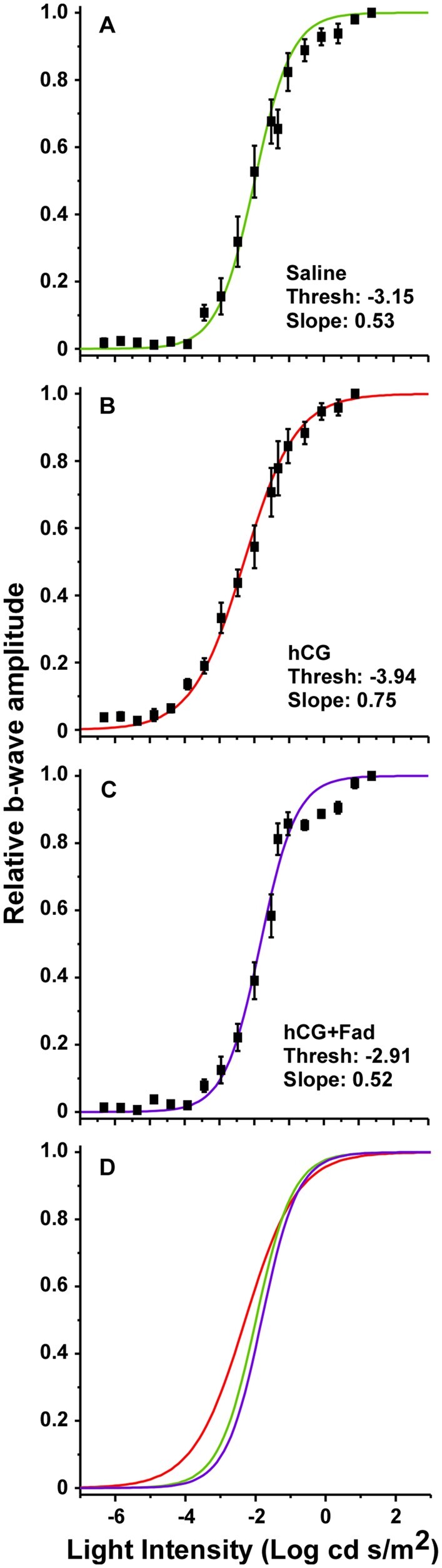
V-log(*I*) curves for the population data in the three treatment groups: (**A**) saline, (**B**) hCG, and (**C**) hCG + fadrozole. Symbols are the mean (±S.E.) for each light intensity step. Mean Boltzmann curves are shown in each panel and in (**D**), revealing modulation by hCG and the lack thereof with the addition of fadrozole.

ERGs exhibited typical a- and b-waves (Fig. 1), the responses of photoreceptors and bipolar cells, respectively (Pugh et al. 1998; Robson and Frishman 1998). In response to dim flashes near visual threshold, a-wave amplitudes were low relative to recording noise. Thus, we utilized the relative b-wave amplitude as a function of stimulus luminance to construct V-Log(*I*) curves (Figs. 1 and 2). From these we determined threshold and slope. For consistency, V-Log(*I*) analysis followed that described in Leslie et al. (2020). The b-wave amplitude was defined as the difference between the average voltage over 20 ms before the light flash and the maximum voltage between 50 and 400 ms after the flash (Rosencrans et al. 2018). The initial stimulus for each procedure consisted of four recordings with no light flash, enabling correction for any DC potential in recordings. The response to each light intensity step was calculated as the average response to four flashes of that intensity. Note that in some cases one of the four responses was removed from the average if there was noise or the electrode came off the cornea. The V-Log(*I*) curve for each individual was normalized to that individual’s maximum b-wave amplitude, resulting in a sigmoidal relative response curve (Miller and Dowling 1970). Response threshold was defined as the light intensity eliciting a response 10% the amplitude of the maximum response (Rosencrans et al. 2018; Leslie et al. 2020). This light intensity was calculated by analyzing each individual V-Log(*I*) curve with a least-squares fit of the standard Boltzmann function:

$$\text{Relative}\;b-\text{wave}\;\text{amplitude}=\frac{A_{1}-A_{2}}{1+e^{\frac{\left(\mathrm{flash}-{\mathrm{flash}}_{0}\right)}{\tau }}}+A_{2}.$$

*A*_1_ is the starting amplitude (0) and *A*_2_ is the ending amplitude (1); flash is the log intensity of each light flash; flash_0_ is the light intensity causing a 50% response; and *τ* is the slope of the function. Statistical significance of differences in treatment group means of the V-Log(*I*) thresholds and slopes was assessed using a one-way ANOVA with Tukey’s HSD test for multiple comparisons.

## Results

**Fig. 3:**
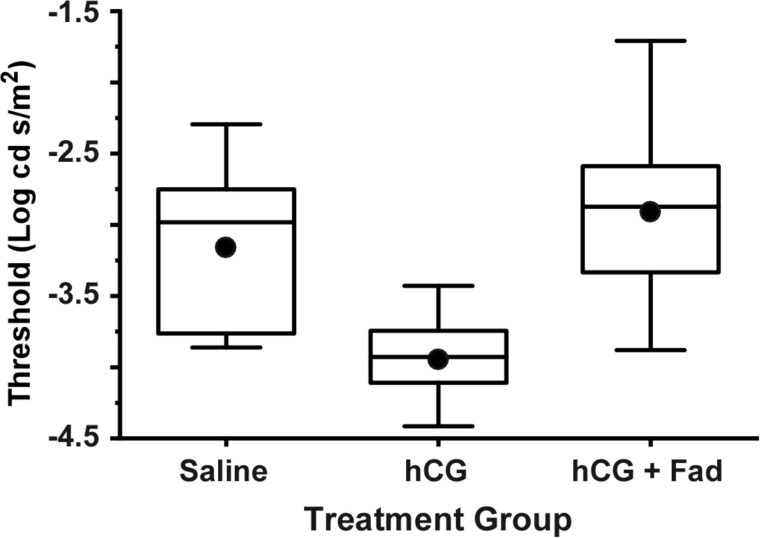
Comparison of ERG b-wave thresholds for the three treatment groups. Means and medians are represented by the filled circles and bold lines, respectively. The lower and upper hinges (i.e., the lower and upper boundaries of each box) mark the first and third quartiles, respectively. Each whisker stretches to the furthest value no further than 1.5 times the interquartile range (distance between the first and third quartiles). hCG treated females had significantly lower mean thresholds (*P* < 0.05) than those treated with saline or hCG + fadrozole. These last two groups did not differ.

A significant main effect of treatment (saline, hCG, hCG + fadrozole) was found for average thresholds (*F*_2,20_ = 6.51; *P* < 0.01). In comparisons between treatment groups, hCG treated individuals had significantly lower thresholds (mean ± S.E.) (−3.94 ± 0.12 Log cd s/m^2^) than the saline injected (−3.15 ± 0.23 Log cd s/m^2^; *P* = 0.04) and the hCG + fadrozole (−2.91 ± 0.26 Log cd s/m^2^; *P* < 0.01) groups. There was no significant difference between average thresholds for the saline and hCG + fadrozole treated animals (*P* = 0.69) (Figs. 2 and 3). Likewise, there was a significant difference in average Boltzmann slope between treatment groups, as determined by one-way ANOVA (*F*_2,20_ = 8.47; *P* < 0.01). The hCG treated group had significantly higher slopes (0.75 ± 0.04) than the saline injected (0.53 ± 0.05; *P* < 0.01) and the hCG + fadrozole (0.52 ± 0.01; *P* < 0.01) groups. There was no significant difference in mean slope between saline and hCG + fadrozole injected groups (*P* = 0.99). Because of the position of the slope term (*τ*) in the Boltzmann equation, higher slope values correspond to more gradual and broader V-Log(*I*) curves, creating greater dynamic range under hCG modulation (Fig. 2). Taken together, the results suggest that the fadrozole treatment prevented hCG modulation of retinal thresholds, implicating estrogen in retinal modulation.

## Discussion

The mechanisms underlying endocrine modulation of the retina during reproduction may be multifold, including hypothalamic hormone, sex steroid, and bioactive lipid components (Butler et al. 2019). This study focused on whether E2 is a modulatory component in túngara frogs. When coupled with fadrozole, the hCG treatment failed to modulate thresholds. Furthermore, the lack of significant differences in both mean threshold and mean slope between the control group and the hCG + fadrozole group indicates that fadrozole, an aromatase inhibitor, successfully blocked retinal modulation by hCG. This suggests that E2 is at least partially responsible for the hCG-induced hormonal modulation of retinal sensitivity previously found in the túngara frogs (Leslie et al. 2020). It should be noted that due to the systemic injection of fadrozole, this was not a specific block of retinal modulation, but rather of E2 production throughout the body. Thus, the source of the E2 is still unknown and may include the eye itself, as aromatase has been localized there and in other sensory structures (Noirot et al. 2009; Maruska and Fernald 2010; Butler et al. 2019).

Estrogens can exert their effects through multiple mechanisms. The classical model involves the nuclear estrogen receptors α and β (ERα and ERβ, respectively), by which estrogen invokes slow, genomic changes (Thomas 2012). Relevant to vision, such receptors have been found in the retinas of humans (Ogueta et al. 1999). In fish retina their expression can be reproductively modulated (Tchoudakova et al. 1999; Friesen et al. 2017b; Butler et al. 2019). Estrogens can also have rapid, nongenomic effects through membrane-bound steroid receptors on the cellular surface (Thomas 2012), such as the G protein-coupled estrogen receptor (GPER, formally known as GPR30), which has been localized in fish, mammalian, avian, and reptilian brains (Brailoiu et al. 2007; Canonaco et al. 2008; Liu et al. 2009; Acharya and Veney 2012; Friesen et al. 2017a; Mangiamele et al. 2017). Its presence in the retina of goldfish has been hypothesized to allow estrogen to rapidly modulate visually guided sexual and social behaviors (Mangiamele et al. 2017); in these fish, injecting males with testosterone or E2 increases their approach behavior to visually cued females (with no other cues present). However, inhibiting aromatase blocks the effect of testosterone administration, indicating that it is E2 that modulates this behavior (Lord et al. 2009). By using ERGs, which only record activity in the retina, our data confirm that modulation is at least localized there, exclusive of central modulatory effects. Nevertheless, it is currently unknown if E2 targets either or both genomic and non-genomic mechanisms to modulate retinal sensitivity.

Estrogenic modulation could explain why female, but not male, túngara frogs experience increased retinal sensitivity when injected with hCG. Because hCG stimulates gonadal release of steroid hormones in both sexes, it is likely that hCG primarily stimulates E2 release in females and androgen release in males (Lynch and Wilczynski 2008; Behrends et al. 2010). Estrogen has traditionally been thought of as a major driver for female-specific sexual behaviors (as opposed to androgens for male-specific sexual behaviors), although the situation can be more complex. For example, E2 has been shown to play a role in activating male reproductive behaviors in avian species such as quail (reviewed in Ball and Balthazart 2004), while testosterone increases auditory thresholds in female green treefrogs (*Hyla cinerea*) (Miranda and Wilczynski 2009). However, studies in frogs and toads largely show strong positive correlations between female sexual behaviors and estrogen and progesterone levels (reviewed in Wilczynski and Lynch 2011), while male sexual behavior seems to be more dependent on the interactions between androgens with other hormones such as prolactin and arginine vasotocin (Moore et al. 2005). In particular, reproductive behaviors of female túngara frogs have been correlated with E2 levels (Lynch and Wilczynski 2005, 2006; Moore et al. 2005; Chakraborty and Burmeister 2009). Considering that assessment of visual cues is a valuable component of female reproductive behavior (Rosenthal et al. 2004; Taylor et al. 2008; Taylor and Ryan 2013), estrogen would be predicted to play a role in reproductive feedback of visual sensitivity. The ethological consequences of this modulation are still untested, however. We propose that by increasing the probability of detecting male vocal sacs in nocturnal habitats (Cummings et al. 2008), modulated (i.e., increased) sensitivity could directly benefit females by improving searching behavior. Nevertheless, further work is needed to conclusively determine the role of estrogen (i.e., its targets and mechanisms) in modulating female retinal sensitivity in these frogs.

## Conclusion

This study provides evidence supporting a major role for E2 in the hormonal modulation of retinal sensitivity in female túngara frogs (Leslie et al. 2020). Administration of fadrozole, an aromatase inhibitor, blocks the modulatory effects of hCG on ERG thresholds in females, leaving those thresholds at untreated, non-reproductive levels. Endocrine modulation of reproductive behavior, including communication and sensory processing, is well known (Yamaguchi and Kelly 2003; Arch and Narins 2009; Leary 2009; Maruska and Sisneros 2015; Caras and Remage-Healey 2016; Wilczynski and Burmeister 2016). Whether or not such modulation is based on changes in mechanisms mediating motivation and/or stimulus sensitivity is often difficult to untangle, as large areas of central and peripheral circuitry may be targets of modulation (Caras 2013). We propose that one area that may isolate sensory modulation is at the sensory receptor organs themselves, where data have accumulated for endocrine effects on stimulus processing within different modalities (Sisneros et al. 2004; Yue et al. 2018; Butler et al. 2019; Perelmuter et al. 2019). To reveal such sensory mechanisms, future work will likely benefit from comparative approaches (Crews and Moore 1986; Adkins-Regan 2005) that choose subject species under selection for context-dependent sensory processing, especially where there are context-dependent changes to the signal-to-noise ratio. The benefits to such approaches could be multifold: not only elucidating modulatory mechanisms, but also informing our understanding of signal evolution through more accurate measurements of receiver processing.

## Author contributions

C.E.L. and W.W. contributed equally to this work. C.E.L., W.W., and H.E.F. conceived of the project, developed the recording and analytical techniques, collected and analyzed the data, and wrote the manuscript. R.F.R. developed the recording and analytical techniques and wrote the manuscript. W.C.G. and N.G.B. oversaw the project, acquired funding and resources, and developed techniques. M.J.R. conceived of and supervised the project, oversaw data analysis and interpretation, and wrote the manuscript.

## Supplementary Material

icab032_Supplementary_Data

## Notes

From the symposium “Sending and receiving signals: Endocrine modulation of social communication” presented at the virtual annual meeting of the Society for Integrative and Comparative Biology, January 3–7, 2020.
