# Supplementary material for: Estrogenic Modulation of Retinal Sensitivity in Reproductive Female Túngara Frogs
Source: Integr Comp Biol. 2021 Apr 26;61(1):231–9. doi: 10.1093/icb/icab032 (PMC8300951; doi:10.1093/icb/icab032)
Supplement: icab032_Supplementary_Data [file icab032_supplementary_data.docx]

**Leslie et al.: Supplementary Table S1**

**Estrogen Targets within Vertebrate Sensory Organs**

| **Species** | **Sensory Modality** | **Sex** | **Anatomical**  **Location** | **Receptor**  **Type(s)** | **Method of**  **Detection** | **Reference** |
| --- | --- | --- | --- | --- | --- | --- |
| **Fish** |  |  |  |  |  |  |
| Midshipman: *Porichthys notatus* | Auditory | Female | Auditory Nerve | ER⍺ | ISH | (Forlano et al. 2005) |
| Midshipman: *Porichthys notatus* | Auditory | Male/ Female | Hair Cells, Saccule | Erβ1/ Erβ2 | IHC/  RT-PCR | (Fergus and Bass 2013) |
|  |  |  |  |  |  |  |
| Burton’s Mouthbrooder: *Astatotilapia burtoni* | Auditory | Male/ Female | Saccule (whole) | ER⍺  Erβa/ Erβb | RT-PCR | (Maruska and Fernald 2010) |
|  |  |  |  |  |  |  |
| Western Mosquitofish: *Gambusia affinis* | Visual | Female | Retina (whole) | ER⍺/ERβ  GPER/GPR30 | RT-PCR | (Friesen et al. 2017) |
| Sailfin Mollies: *Poecilia latipinna* | Visual | Female | Retina (whole) | ER⍺/ERβ  GPER/GPR30 | RT-PCR | (Friesen et al. 2017) |
| Goldfish: *Carassius auratus* | Visual | Male/ Female | Retina (whole) | GPER/GPR30 | RT-PCR | (Mangiamele et al. 2017) |
| Burton’s Mouthbrooder: *Astatotilapia burtoni* | Visual | Female | Retina (whole) | ER⍺ | RT-PCR | (Butler et al. 2019) |

| **Mammals** |  |  |  |  |  |  |
| --- | --- | --- | --- | --- | --- | --- |
| Mouse:  *Mus musculus* | Auditory | Male/ Female | Inner Hair Cells, Outer Hair Cells, Spiral Ganglion,  Type 1 cells,  Crista Ampullaris, Dark Cells, Endolymphatic Sac Epithelial Cells,  Stria Vascularis | ER⍺/ERβ | IF | (Motohashi et al. 2010) |
| Mouse:  *Mus musculus* | Olfactory | Female | Olfactory Epithelial Cells | GPER/GPR30 | IF | (Kanageswaran et al. 2016) |
| Mouse:  *Mus musculus* | Visual | Male | Retina (Inner Nuclear Layer: INL; Ganglion Cell Layer: GCL) | GPER/GPR30 | IF | (Jiang et al. 2019) |
| Human:  *Homo sapiens* | Auditory | Male/ Female | Type 1 Cells*,  Marginal Cells** | ER⍺/ERβ | IHC | (Stenberg et al. 2001) |
| **Avian Reptiles** |  |  |  |  |  |  |
| Zebra Finch: *Taeniopygia guttata* | Auditory | Male/ Female | Inner Hair Cells,  Outer Hair Cells,  Supporting Cells | ER⍺ | IF | (Noirot et al. 2009) |

*ER⍺ only, ** ERβ only

*In situ* hybridization (ISH), Immunohistochemistry (IHC), Immunofluorescence (IF), Reverse Transcriptase Polymerase Chain Reaction (RT-PCR)

Butler JM, Whitlow SM, Rogers LS, Putland RL, Mensinger AF, Maruska KP. 2019. Reproductive state-dependent plasticity in the visual system of an african cichlid fish. Horm Behav. 114:104539.

Fergus DJ, Bass AH. 2013. Localization and divergent profiles of estrogen receptors and aromatase in the vocal and auditory networks of a fish with alternative mating tactics. J Comp Neurol. 521(12):2850-2869.

Forlano PM, Deitcher DL, Bass ARH. 2005. Distribution of estrogen receptor alpha mrna in the brain and inner ear of a vocal fish with comparisons to sites of aromatase expression. J Comp Neurol. 483(1):91-113.

Friesen CN, Ramsey ME, Cummings ME. 2017. Differential sensitivity to estrogen-induced opsin expression in two poeciliid freshwater fish species. Gen Comp Endocrinol. 246:200-210.

Jiang M, Ma X, Zhao Q, Li Y, Xing Y, Deng Q, Shen Y. 2019. The neuroprotective effects of novel estrogen receptor gper1 in mouse retinal ganglion cell degeneration. Exp Eye Res. 189:107826.

Kanageswaran N, Nagel M, Scholz P, Mohrhardt J, Gisselmann G, Hatt H. 2016. Modulatory effects of sex steroids progesterone and estradiol on odorant evoked responses in olfactory receptor neurons. PLoS One. 11(8):e0159640.

Mangiamele LA, Gomez JR, Curtis NJ, Thompson RR. 2017. Gper/gpr30, a membrane estrogen receptor, is expressed in the brain and retina of a social fish (*Carassius auratus*) and colocalizes with isotocin. J Comp Neurol. 525(2):252-270.

Maruska KP, Fernald RD. 2010. Steroid receptor expression in the fish inner ear varies with sex, social status, and reproductive state. BMC Neurosci. 11:58.

Motohashi R, Takumida M, Shimizu A, Konomi U, Fujita K, Hirakawa K, Suzuki M, Anniko M. 2010. Effects of age and sex on the expression of estrogen receptor alpha and beta in the mouse inner ear. Acta Otolaryngol. 130(2):204-214.

Noirot IC, Adler HJ, Cornil CA, Harada N, Dooling RJ, Balthazart J, Ball GF. 2009. Presence of aromatase and estrogen receptor alpha in the inner ear of zebra finches. Hear Res. 252(1-2):49-55.

Stenberg AE, Wang H, Fish J, 3rd, Schrott-Fischer A, Sahlin L, Hultcrantz M. 2001. Estrogen receptors in the normal adult and developing human inner ear and in turner's syndrome. Hear Res. 157(1-2):87-92.
